# Feedback on and knowledge, attitude, and skills at the end of pharmacology practical sessions

**DOI:** 10.3352/jeehp.2011.8.12

**Published:** 2011-11-30

**Authors:** P. Ravi Shankar, Nisha Jha, Omi Bajracharya, Sukh B Gurung, Kundan K. Singh

**Affiliations:** Department of Clinical Pharmacology and Therapeutics, KIST Medical College, Lalitpur, Kathmandu, Nepal.

**Keywords:** Attitudes, Effectiveness, Impact, Knowledge, Nepal, Pharmacology, Prescribing, Skills

## Abstract

Concern has been raised about inadequate pharmacology teaching in medical schools and the high incidence of prescribing errors by doctors in training. Modifications in pharmacology teaching have been carried out in many countries. The present study was carried out using a semi-structured questionnaire to obtain students' perceptions of their knowledge, attitudes, and skills with regard to different subject areas related to rational prescribing at the end of two-year activity-based pharmacology practical learning sessions in a private medical school in Nepal. The effectiveness of the sessions and strengths and suggestions to further improve the sessions were also obtained. The median total knowledge, attitude, skills and overall scores were calculated and compared among different subgroups of respondents. The median effectiveness score was also calculated. Eighty of the 100 students participated; 37 were male and 43 female. The median knowledge, attitude, and skills scores were 24, 39, and 23, respectively (maximum scores being 27, 45, and 36). The median total score was 86 (maximum score being 108). The effectiveness score for most subject areas was 3 (maximum 4). The strengths were the activity-based nature of the session, use of videos and role-plays, and repeated practice. Students wanted more sessions and practice in certain areas. They also wanted more resources and an internet connection in the practical room. The skills scores were relatively low. The immediate impact of the sessions was positive. Studies may be needed to assess the long term impact. Similar programs should be considered in other medical schools in Nepal and other developing countries.

## INTRODUCTION

Traditional teaching-learning in pharmacology has focused more on theoretical knowledge of medicines than on using them in practice. In most countries, pharmacology is taught during the early, basic science years of the course and students memorize information about drugs without a direct clinical context [1]. Pharmacology textbooks are also written centered on drugs and the reason why a particular treatment is chosen often remains unclear. Teaching students to choose medicines based on scientific principles in a safe and effective manner is a major challenge for medical schools [2]. Problem-based pharmacotherapy teaching in undergraduate medical education based on a national essential medicine list and standard treatment guidelines has been recommended as a key intervention to improve prescribing [3].

### Studies in developed nations

A recent article mentioned the high incidence of drug prescribing errors and attributed it mainly to inadequate pharmacology teaching [4]. The pervasive and increasingly close relationship between doctors and the pharmaceutical industry influences prescribing habits and the drug education of doctors. In developed nations like the United Kingdom (UK), a core curriculum in prescribing and therapeutics has been developed for medical schools [5]. A study in Australia demonstrated that interns about to commence clinical practice had significant deficits in their prescribing skills [6]. Most interns were in favor of more teaching of clinical pharmacology in medical schools.

In the Netherlands, preclinical medical students were taught to select, prescribe, and evaluate a drug regimen [7]. Preclinical learning in context led to the use of more rational prescribing modalities by students during their internal medicine clerkships. Problem-oriented or problem-based learning (PBL) in pharmacology has been recommended to improve the prescribing skills of doctors. In a university in Slovakia, a hybrid of PBL and lectures was used to teach medical pharmacology, which improved the decision-making skills of future prescribers [8]. Modifications of PBL with students divided into small groups and each group being further divided into clusters with each cluster working on a particular problem [9] and team-based learning [10] have been used to conduct PBL for large groups of students. A mulitcenter randomized controlled study showed that the exercise of creating a personal formulary was effective in teaching rational prescribing [11]. There is a high probability that a large number of drugs chosen in the formulary will be essential drugs. In the United States, a therapeutics curriculum emphasizes pharmacovigilance and drug safety [12].

### Studies in developing countries

In a study conducted in a medical school in Nigeria, students wanted teaching using audiovisual aids and inclusion of clinical pharmacology in lectures [13]. They wanted inclusion of seminars and group discussion and felt pharmacology teaching should be more clinically oriented. In India, most students were satisfied with all teaching methods except lectures, seminars, and pharmacy exercises [14]. Students preferred tutorials, short answer questions, and revision classes. They wanted more clinical pharmacology and bedside teaching. In Mexico, pharmacology is taught during the second year of the course covering both basic aspects and clinical pharmacology. However, it was found that training was sometimes inadequate and drugs were prescribed irrationally [15]. At BP Koirala Institute of Health Sciences, Dharan, Nepal, an exercise on drug utilization was used to introduce students to problems with use of antibiotics and inculcate proper prescribing of antibiotics [16]. A sequential decision making process has been recommended to orient students towards rational therapeutics [17].

In Nepal, pharmacology is taught in an integrated organ system-based manner along with other basic science subjects and community medicine during the first two years of the undergraduate medical (MBBS) course. At KIST Medical College (KISTMC), Lalitpur, Nepal, the department of clinical pharmacology and therapeutics is committed to teaching students to use essential medicines rationally. Students work in groups of ten students each and facilitators rotate among groups [18]. Students learn about essential medicines, analyze prescribing using World Health Organization/International Network for Rational Use of Drugs (WHO/INRUD) prescribing indicators, select the personal drug (P-drug) for a particular disease condition, verify the suitability of the selected P-drug for a particular patient, write the prescription (P-drug selection process), and communicate non-drug and drug measures to manage the condition with a simulated patient. Students critically analyze drug advertisements and other promotional materials, solve clinical problems, learn to optimize time spent with medical representatives (MRs), and become familiar with independent sources of medicine information. Student opinion about the sessions was obtained previously and was positive [18]. Detailed feedback about the knowledge, attitudes, and skills of students on completing the practical sessions and the effectiveness of the sessions with regard to particular subject areas/learning objectives has not been obtained previously. The subject areas identified were essential medicines, the P-drug selection process, social issues in the use of medicines, responding appropriately to pharmaceutical promotion, using independent sources of medicine information, using antibiotics rationally, analyzing prescribing using WHO/INRUD indicators, communicating with a simulated patient, and reporting adverse drug reactions. Hence, the present study was conducted with the following objectives:

1. Obtain basic demographics of student respondents
2. Obtain respondents' perception of their knowledge, attitudes, and skills at the end of the sessions
3. Note respondents' view of the effectiveness of the sessions with respect to particular learning objectives
4. Note the association of median knowledge, attitudes, skills, and effectiveness scores with demographic characteristics, if any, and
5. Obtain strengths, unique features, and suggestions for improvement of the sessions with respect to particular learning objectives.

## MATERIALS AND METHODS

The study was conducted using a semi-structured questionnaire during early September 2011 when the practical sessions for second year students were about to finish. Demographic information including gender, method of financing of medical education, and father's and mother's occupations were noted.

The study was approved by the Institutional Review Board of KIST Medical College, Nepal. Participants were explained the aims and objectives of the study and invited to participate. Written informed consent was obtained from all participants. The questionnaire used is shown in the Appendix.

Nine subject areas/learning objectives of the pharmacology practical sessions were delineated as stated previously. Respondents' knowledge, attitudes, and skills in these subject areas were noted at the end of the practical sessions conducted over a two-year period. The effectiveness of the sessions with respect to these subject areas was also noted.

For knowledge, the following scoring system was adopted. "No idea" was given the score 1; "a vague idea," the score 2; and "a clear idea," the score 3. The median knowledge score for each area and the total knowledge score were calculated. For attitude, the scoring system adopted was "strongly disagree (1)", "disagree (2)", "neutral (3)", "agree (4)", and "strongly agree (5)". The total attitude scores were calculated. Certain statements were negative and their scores were reversed to calculate the total attitude score. For skills, "not confident" was given the score 1; "somewhat confident," 2; "very confident," 3; and "will be able to do independently in the future," 4. The total skills scores were calculated. The total knowledge, attitude, and skills (KAS) scores (overall scores) were calculated by adding the total knowledge, total attitude, and total skills scores. The normality in distribution of the scores was noted using a one sample Kolmogorov-Smirnov test. The distribution was not normal and non-parametric tests were used to compare the median total scores among different categories of respondents. A P-value less than 0.05 was taken as statistically significant.

The effectiveness of the sessions for each learning objective was also studied. "Not effective" was given the score 1; "somewhat effective," 2; "effective," 3; and "very effective," 4. The median effectiveness score was calculated and compared among subgroups of respondents using the appropriate non-parametric tests.

Participants were asked to mention one strength or unique feature of the session with respect to the particular subject area and also to provide one suggestion to further improve the sessions with respect to a particular subject area. The frequency of responses was noted. Common responses were tabulated.

## RESULTS

Eighty of the 100 second year students (80%) participated; thirty-seven (46.2%) were male and 43 (53.8%) were female. Ten were scholarship students, 64 were self-financing, and six did not provide this information. Twenty-three students (29.1%) had fathers in health-related professions. Fifty-five mothers (68.8%) were housewives, 20 (25%) were working in non-health related professions, and four were in health related professions; one student did not provide this information.

Table 1 shows the median knowledge, attitude, and skills scores according to different subject areas emphasized during the pharmacology practical sessions. The median total knowledge score was 24 (maximum score 27). The median total attitude and skills scores were 39 and 23, respectively. The maximum possible scores were 45 and 36, respectively. The median total combined score of knowledge, skills and attitudes (KAS score) was 86 (maximum score being 108).

Table 2 shows the median scores according to personal characteristics of the respondents. The median skills score was higher among male respondents and among respondents whose mother was a housewife. Table 3 shows the median effectiveness scores according to different subject areas covered during pharmacology practical sessions. The effectiveness scores for most subject areas were 3 (maximum score 4).

The strengths/characteristic features of the session under each subject heading were investigated. With regard to essential medicines, the strengths were that a clear concept was provided (14 respondents), they obtained knowledge about essential medicines in Nepal (8 respondents), they understood the importance of essential medicines (6 respondents), and they came to recognize the importance of essential medicines in rural Nepal (4 respondents). In the subject area of the P-drug selection process, the strengths were that the process was done repeatedly (12 respondents), students were introduced to the process of objective drug selection (12 respondents), students understood how to prescribe specific drugs for a specificdisease (11 respondents), students were confident in selecting P-drugs (10 respondents), and students learned to choose drugs according to the patient's condition (6 respondents). With regard to social issues in the use of medicines, the benefits were interesting role plays, students learnt to use drugs according to the patient's background, they were aware of social issues in the context of Nepal, and the issues discussed would prepare them for future practice. Regarding the subject of pharmaceutical promotion, 12 respondents opined that videos were used effectively, 10 respondents stated they were confident in dealing with promotion, nine stated that the training would help to deal with MRs, and seven stated that the lessons would be useful in future practice. They were also confident that they could resist the promotional activities of the industry. With regard to independent sources of medicine information, the respondents noted that they were aware of different independent sources and were aware that not all sources were reliable.

Coming to the topic the rational use of antibiotics, 10 respondents stated that the sessions clarified about antibiotic use in practice, nine stated that rational use is vital to prevent resistance, seven said they learned to use antibiotics cautiously, and six stated they learned not to use antibiotics for viral infections and the common cold. With regard to WHO/INRUD indicators, the points stated were that students learned to write proper prescriptions avoiding common errors, they were made aware of standards of prescription, and the exercise was very useful for checking prescribing patterns in a particular health facility in an area. With regard to the activity of communicating drug and non-drug information with a simulated patient, the comments were that the activity taught students how to deal with patients (20 respondents), improved their communication skills (10 respondents), will be helpful in the future (9 patients), and the skills were practiced repeatedly (7 respondents). With regard to reporting adverse drug reactions (ADRs), the comments were that the activities taught students the process of reporting, that ADR reporting improves drug safety, it improves patient care, and the exercise of creating their own ADR reporting form was effective.

Table 4 shows the common suggestions for further improving the sessions. Students wanted a larger number of sessions in certain areas.

## DISCUSSION

Student participation in the study was high. The median knowledge and attitude scores were high but the skills scores were comparatively low. The median scores did not vary significantly according to demographic characteristics. The effectiveness scores of the session with regard to each learning objective were satisfactory. The strengths of the sessions overall were clear concepts, repeated practice, and use of active learning strategies. Students wanted more sessions and more practice in certain learning areas.

### Essential medicines

Problems in this subject area include difficulty in obtaining a copy of the essential drug list of Nepal and in allotting adequate time to the topic because of other subject areas to be discussed. During this exercise we also teach students about generic medicines and brand name medicines, product and process patents, TRIPS, and the Doha declaration, among other topics.

### P-drugs

Students develop a set of standard treatments for common diseases and a set of first choice drugs called personal or P-drugs using standard treatment guidelines, formularies, textbooks, and other independent sources of medicine information [19]. The exercise of P-drug selection is being conducted at the Manipal College of Medical Sciences (MCOMS), Pokhara, Nepal [20]. At KISTMC, the exercise has been conducted for the last three years [18]. In both institutions, the method described by Joshi and Jayawickramarajah [17] is followed. The exercise occupies an important place in the practical assessment in pharmacology [21].

### Social issues

A medical humanities module called Sparshanam is conducted for all first year students [22]. During the pharmacology practical, we use role plays and small group activities to explore issues like patient preference for injections, not using antibiotics properly, stopping antibiotics on feeling better, preference for more expensive medicines, patient misconceptions about disease and medicines, and patient difficulty in accessing medicines and health services, among others.

### Pharmaceutical promotion

Pharmaceutical promotion is becoming an important factor influencing the use of medicines, even in developing countries like Nepal [23]. Students learn to critically analyze drug advertisements and promotional material, to optimize time spent with MRs, and about disease mongering. Teaching students to understand and respond to pharmaceutical promotion is a challenge for medical educators in south Asia [24]. A survey found over 60% of institutions worldwide had used small group discussions in tutorials or workshops [25] to teach about pharmaceutical promotion to their students. Recently the WHO and Health Action International (HAI) have produced a manual entitled *Understanding and responding to pharmaceutical promotion: a practical guide* [26] to be used in conducting modules on pharmaceutical promotion for medical and pharmacy students.

### Independent sources of medicine information

Using independent sources of medicine information is an important skill for all doctors. In Nepal, drug information centers are available in many teaching hospitals. At MCOMS, Pokhara, the pharmacology department runs a drug information center in the teaching hospital to provide objective, unbiased information about drugs and therapeutics to prescribers [27], which is also used to train medical, nursing, and pharmacy students. At KISTMC, students use sources like the Nepalese national formulary, Patan hospital formulary, and British national formulary during sessions. Students learn to access and assess the quality of health information on the internet. An internet connection in the laboratory, as suggested, would be useful in teaching about online information sources.

### Rational use of antibiotics

Rational use of antibiotics is becoming an important issue. Improper and irrational use of antibiotics is an important factor responsible for antibiotic resistance. We also conduct an exercise analyzing the rationality of prescriptions, and students correct improper prescriptions. Students learn to select antibiotics using the criteria of efficacy, safety, cost, and suitability. They also learn not to use antibiotics in predominantly viral infections like the common cold and upper respiratory infections.

### Prescribing indicators

Prescribing indicators have been widely used to assess prescribing, especially in primary health facilities. A study conducted in a medical school in Turkey showed that prescription audit sessions can be an easy and effective method for reinforcing prescribing skills acquired through rational prescribing education [28]. This exercise could be improved by having students analyze prescriptions at government health facilities during their community diagnosis posting.

### Communication skills

Standardized patients have been used to teach clinical skills to medical students in ambulatory care settings [29]. They have been widely used to assess communication skills among both medical and nursing students [30]. Their use in both learning and assessment is widespread. We assess students in communication skills at an objective structured practical examination station using a simulated patient and a structured checklist [20].

### Pharmacovigilance

Teaching students about ADR reporting and pharmacovigilance is important because as future doctors they are important for the success of an ADR reporting program. At MCOMS, students are taught about pharmacovigilance and an exercise in designing an ADR reporting form has been conducted [31]. A study conducted in the UK and published in 2004 found that only 57% of medical schools featured the yellow card scheme, the spontaneous reporting scheme in the UK in their curriculum [32]. Teaching about pharmacovigilance should sensitize students about the importance of ADR reporting, motivate them to report, and equip them with the skills and knowledge to successfully do so.

Pharmaceutical calculations have been covered for a few cohorts, but the learning has not been systematic. We are planning to introduce this in future sessions.

In 2009, a systematic review of educational interventions to improve prescribing by junior doctors and medical students was conducted [33]. The authors concluded that most studies had only small numbers of participants and had methodological flaws. The WHO 'Guide to Good Prescribing' is the only widely used model that has been shown to improve prescribing, and it needs further development. Robust methods of assessment are required to assess the impact of teaching interventions. Studies on the long term impact of educational interventions are lacking, especially in developing nations.

The strength of the study was the high response rate. The study also had limitations. The questionnaire and the scoring system were developed by the authors in consultation with a statistician who also has expertise in medical education. However, the questionnaire was not validated. The free text comments about strengths/unique features of the practical session and suggestions for further improvement were few. Student perceptions were not triangulated with data obtained from other sources.

The overall student response was positive. The knowledge and attitude scores were high. The skills scores were low. The students may require more practice and time to further develop their skills and they will put these skills into practice during their internship training. The authors have shown that it is possible to conduct a practical learning program in pharmacology using minimal resources in a developing country. The study shows the immediate impact of the program has been positive. Studies are needed to assess the long term impact. Prescribing during the internship and in future practice will be the true measure of the success of the program. Students should put their knowledge into practice during future clinical training. Similar programs should be considered in other medical schools in Nepal and other developing countries.

## Figures and Tables

**Table 1:**
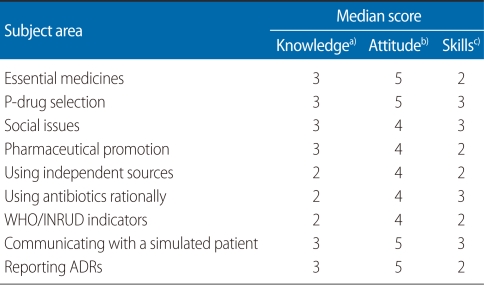
Median knowledge, attitude, and skills scores in each subject area

WHO, World Health Organization; INRUD, International Network for the Rational Use of Drugs; ADR, adverse drug reaction. Maximum score ^a)^3, ^b)^5, ^c)^4.

**Table 2:**
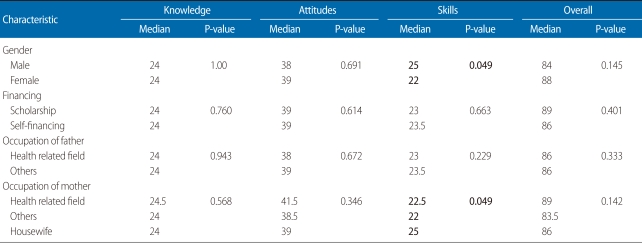
Median knowledge, attitude, skills, and overall scores according to respondent's personal characteristics

**Table 3:**
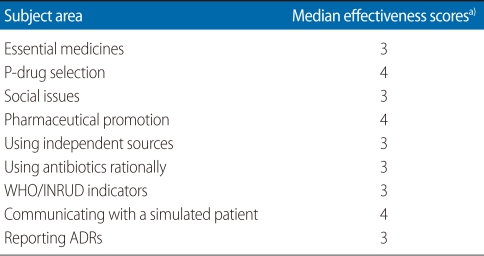
Median effectiveness scores according to personal characteristics of respondents

WHO, World Health Organization; INRUD, International Network for the Rational Use of Drugs; ADR, adverse drug reaction. ^a)^Maximum score 4.

**Table 4:**
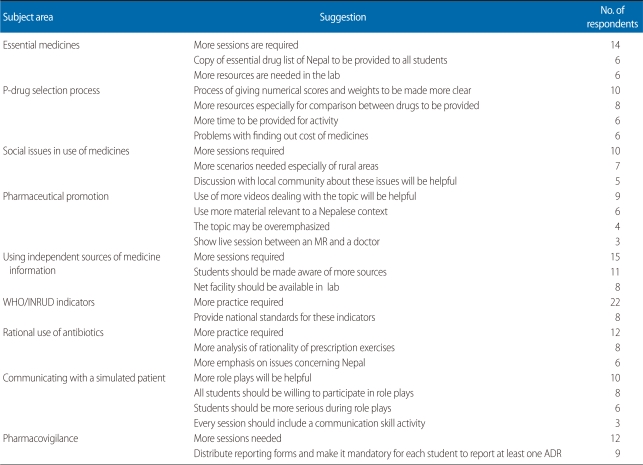
Common suggestions to further improve the sessions with regard to a particular subject area

WHO, World Health Organization; INRUD, International Network for the Rational Use of Drugs; ADR, adverse drug reaction.

## References

[1] Hudec R, Tisonova J, Bozekova L, Wawruch M, Kriska M, Kristova V (2009). Modified problem-based learning in pharmacology. Bratisl Lek Listy.

[2] Flockhart DA, Usdin Yasuda S, Pezzullo JC, Knollmann BC (2002). Teaching rational prescribing: a new clinical pharmacology curriculum for medical schools. Naunyn Schmiedebergs Arch Pharmacol.

[3] Laing R, Hogerzeil H, Ross-Degnan D (2001). Ten recommendations to improve use of medicines in developing countries. Health Policy Plan.

[4] Gwee MC (2009). Teaching of medical pharmacology: the need to nurture the early development of desired attitudes for safe and rational drug prescribing. Med Teach.

[5] Maxwell S, Walley T, BPS Clinical Section Committee (2003). Teaching safe and effective prescribing in UK medical schools: a core curriculum for tomorrow's doctors. Br J Clin Pharmacol.

[6] Hilmer SN, Seale JP, Le Couteur DG, Crampton R, Liddle C (2009). Do medical courses adequately prepare interns for safe and effective prescribing in New South Wales public hospitals?. Intern Med J.

[7] Richir MC, Tichelaar J, Stanm F, Thijs A, Danner SA, Schneider AJ, de Vries TP (2008). A context-learning pharmacotherapy program for preclinical medical students leads to more rational drug prescribing during their clinical clerkship in internal medicine. Clin Pharmacol Ther.

[8] Tisonova J, Hudec R, Szalayova A, Bozekova L, Wawruch M, Lassanova M, Vojtko R, Jezova D, Kristova V, Kriska M (2005). Experience with problem oriented teaching in pharmacology. Bratisl Lek Listy.

[9] Kingsbury MP, Lymn JS (2008). Problem-based learning and larger student groups: mutually exclusive or compatible concepts: a pilot study. BMC Med Educ.

[10] Zgheib NK, Simaan JA, Sabra R (2011). Using team-based learning to teach clinical pharmacology in medical school: student satisfaction and improved performance. J Clin Pharmacol.

[11] De Vries TP, Daniels JM, Mulder CW, Groot OA, Wewerinke L, Barnes KI, Bakathir HA, Hassan NA, Van Bortel L, Kriska M, Santoso B, Sanz EJ, Thomas M, Ziganshina LE, Bezemer PD, Van Kan C, Richir MC, Hogerzeil HV (2008). Should medical students learn to develop a personal formulary? An international, multicentre, randomised controlled study. Eur J Clin Pharmacol.

[12] Naritoku DK, Faingold CL (2009). Development of a therapeutics curriculum to enhance knowledge of fourth-year medical students about clinical uses and adverse effects of drugs. Teach Learn Med.

[13] Oshikoya KA, Bello JA, Ayorinde EO (2007). Medical student's view on the methods of teaching pharmacology at the Lagos State University College of Medicine, Nigeria. Nig Q J Hosp Med.

[14] Badyal DK, Bala S, Kathuria P (2010). Student evaluation of teaching and assessment methods in pharmacology. Indian J Pharmacol.

[15] Rodriguez-Carranza R, Vidrio H, Campos-Sepulveda E (2008). The teaching of pharmacology in medical schools: current status and future perspectives. Gac Med Mex.

[16] Das BP, Sethi A, Nutan K, Gunjan (2005). Teaching exercise of drug utilization by medical students. JNMA J Nepal Med Assoc.

[17] Joshi MP, Jayawickramarajah PT (1996). A problem-orientated pharmacotherapy package for undergraduate medical students. Med Teach.

[18] Shankar PR, Jha N, Bajracharya O, Shrestha R, Thapa HS (2010). Teaching pharmacology at a Nepalese medical school: the student perspective. Australas Med J.

[19] Hogerzeil HV, Barnes KI, Henning RH, Kocabasoglu YE, Moller H, Smith AJ, Summers RS, de Vries TP (2001). Teachers' guide to good prescribing.

[20] Shankar PR, Palaian S, Gyawali S, Mishra P, Mohan L (2007). Personal drug selection: problem-based learning in pharmacology: experience from a medical school in Nepal. PLoS One.

[21] Shankar PR, Gurung SB, Jha N, Bajracharya O, Ansari SR, Thapa HS (2010). Practical assessment in Pharmacology at a new Nepalese medical school. J Clin Diagn Res.

[22] Shankar PR, Piryani RM, Upadhyay-Dhungel K (2011). Student feedback on the use of paintings in Sparshanam, the Medical Humanities module at KIST Medical College, Nepal. BMC Med Educ.

[23] Giri BR, Shankar PR (2005). Learning how drug companies promote medicines in Nepal. PLoS Med.

[24] Shankar PR, Piryani RM (2009). Medical education and medical educators in South Asia: a set of challenges. J Coll Physicians Surg Pak.

[25] Mintzes B (2005). Educational initiatives for medical and pharmacy students about drug promotion: an international cross-sectional survey.

[26] Mintzes B, Mangin D, Hayes L (2011). Understanding and responding to pharmaceutical promotion: a practical guide.

[27] Shankar PR (2006). Pharmacology at the Manipal College of Medical Sciences, Pokhara, Nepal: new roles and new challenges. Internet J Pharmacol.

[28] Akici A, Goren MZ, Aypak C, Terzioglu B, Oktay S (2005). Prescription audit adjunct to rational pharmacotherapy education improves prescribing skills of medical students. Eur J Clin Pharmacol.

[29] Myung SJ, Kang SH, Kim YS, Lee EB, Shin JS, Shin HY, Park WB (2010). The use of standardized patients to teach medical students clinical skills in ambulatory care settings. Med Teach.

[30] Ryan CA, Walshe N, Gaffney R, Shanks A, Burgoyne L, Wiskin CM (2010). Using standardized patients to assess communication skills in medical and nursing students. BMC Med Educ.

[31] Shankar PR, Subish P (2006). Designing a spontaneous adverse drug reaction reporting form: an exercise for medical students. Int J Risk Saf Med.

[32] Cox AR, Marriott JF, Wilson KA, Ferner RE (2004). Adverse drug reaction teaching in UK undergraduate medical and pharmacy programmes. J Clin Pharm Ther.

[33] Ross S, Loke YK (2009). Do educational interventions improve prescribing by medical students and junior doctors? A systematic review. Br J Clin Pharmacol.

